# Mechanistic Study of Fast Performance Decay of PtCu Alloy-based Catalyst Layers for Polymer Electrolyte Fuel Cells through Electrochemical Impedance Spectroscopy

**DOI:** 10.3390/ma16093544

**Published:** 2023-05-05

**Authors:** Maximilian Grandi, Matija Gatalo, Ana Rebeka Kamšek, Gregor Kapun, Kurt Mayer, Francisco Ruiz-Zepeda, Martin Šala, Bernhard Marius, Marjan Bele, Nejc Hodnik, Merit Bodner, Miran Gaberšček, Viktor Hacker

**Affiliations:** 1 Institute of Chemical Engineering and Environmental Technology, Graz University of Technology, Inffeldgasse 25/C, 8010 Graz, Austria; 2 Department of Materials Chemistry, National Institute of Chemistry, Hajdrihova 19, 1000 Ljubljana, Slovenia; 3 Department of Analytical Chemistry, National Institute of Chemistry, Hajdrihova 19, 1000 Ljubljana, Slovenia

**Keywords:** PEFC, catalyst layer, platinum–copper, degradation, ionomer, electrochemical impedance spectroscopy, membrane electrode assembly

## Abstract

In the past, platinum–copper catalysts have proven to be highly active for the oxygen reduction reaction (ORR), but transferring the high activities measured in thin-film rotating disk electrodes (TF-RDEs) to high-performing membrane electrode assemblies (MEAs) has proven difficult due to stability issues during operation. High initial performance can be achieved. However, fast performance decay on a timescale of 24 h is induced by repeated voltage load steps with H_2_/air supplied. This performance decay is accelerated if high relative humidity (>60% RH) is set for a prolonged time and low voltages are applied during polarization. The reasons and possible solutions for this issue have been investigated by means of electrochemical impedance spectroscopy and distribution of relaxation time analysis (EIS–DRT). The affected electrochemical sub-processes have been identified by comparing the PtCu electrocatalyst with commercial Pt/C benchmark materials in homemade catalyst-coated membranes (CCMs). The proton transport resistance (*R*_pt_) increased by a factor of ~2 compared to the benchmark materials. These results provide important insight into the challenges encountered with the de-alloyed PtCu/KB electrocatalyst during cell break-in and operation. This provides a basis for improvements in the catalysts’ design and break-in procedures for the highly attractive PtCu/KB catalyst system.

## 1. Introduction

In the race to cut the world’s greenhouse gas emissions, the transition towards renewable energy solutions is crucial. An important piece of the puzzle is the water cycle, in conjunction with water splitting and polymer electrolyte fuel cells (PEFCs), which are assumed to play a highly important role in the transport sector of the future [1,2]. With the large-scale commercialization of PEFC already in sight, the high cost remains a major challenge for PEFC technology. The electrocatalyst represents one of the two highest cost contributions to stack manufacturing [3,4]. While platinum group metal-free (PGM-free) catalysts [5] might represent a solution in the more distant future, inherently sluggish oxygen reduction reaction (ORR) currently still requires too large an amount of platinum (Pt). In this sense, Pt-based nano-alloys (Pt-M) with less expensive 3D transition metals (M = Co, Ni), are the most commercially advanced solution to quickly reach the production phase for PEFC [6,7,8] and reduce costs [9,10,11,12,13,14,15,16,17,18,19,20]. In this group of compounds, PtCu stands out because of its even higher activity towards ORR [21,22] as well as other electrochemical reactions, e.g., alcohol oxidation [23]. Currently however, the biggest obstacle for PtCu compounds seems to be the gap between the remarkable activities measured on the laboratory scale with the thin film rotating disk electrode method (TF-RDE) and the industrially relevant membrane electrode assembly (MEA) [6,24,25,26]. Testing at the single-cell level has revealed an improved activity towards ORR in the kinetically controlled region at 0.9 V_RHE_ with respect to a Pt/C reference. However, after exposure to lower voltages (and higher current densities; HCDs), the performance has been shown to decay quickly [6,27]. The consensus in the literature is that the PtCu alloy system suffers from detrimental negative effects from dissolved Cu ions [6,24,25,26,27]. Such a possibility was identified both under half-cell [24] and single-cell conditions [25]. On the cathode side, Cu can interact with the Pt surface under PEFC operation-relevant potentials via underpotential deposition (UPD) and partly block the favorable four-electron pathway, causing peroxide formation and thus negatively affecting ORR [24]. This can result in durability issues due to the promotion of the Fenton reaction by Cu [28,29], leading to high hydroxyl radical concentrations within the electrolyte. Migration to the anode could cause proton starvation of the cathode due to Cu plating of the Pt/C catalyst and thus blocking of the hydrogen oxidation reaction (HOR) on the anode [24,25]. The effects of Cu ions can also be influenced via operating conditions such as high relative humidity and high current [30], which promote metal dissolution or result in migration of the metal ions. While these processes all happen after several thousand CV cycles with nitrogen on the cathode and with upper vertex potentials higher than the open circuit potential at H_2_/air, Falina et al. [26] and Gatalo et al. [6] observed performance degradation without extensive voltage cycling. Furthermore, an increase in the amount of dissolved nickel and copper at low voltages/high currents has been confirmed using a flow-through electrochemical half-cell coupled with ICP–MS analysis of the electrolyte [6]. The studies however remained inconclusive on the mechanism of the damage under application-oriented operating conditions with H_2_/air supplied and no extensive voltage cycling with nitrogen supplied on the cathode, low cell potentials and short times at open cell potential (OCP). Questions are open as to which component is damaged and where the Cu ions are localized, since the migration of Cu ions is undoubtedly influenced by the water flux through the membrane electrode assembly. The net direction and magnitude of water flux through the membrane is highly dependent on the temperature [31], proton current density, and the water concentration gradient between anode and cathode [31,32,33]. At high current densities, constant temperature and relative humidity, and low water concentration gradient between anode and cathode, water moves from anode to cathode, while at low current densities (e.g., during voltage cycling with nitrogen on the cathode), the direction of water flux could be inverse or at least much lower, as showcased by the water transport equations of M. Hinaje et al. (Equation (1)) [33].

(1) $${\vec{N}}_{H_{2}O}^{m}=\frac{n_{d}}{F}*\vec{J}-D_{H_{2}O}^{m}*\vec{\nabla }C_{H_{2}O}$$

This means that under operating conditions closer to the application, the possibility is high that dissolved Cu could not reach the anode as observed after extensive voltage cycling with nitrogen.

A very effective way for the analysis of electrochemical processes and resistance contributions in the MEA is electrochemical impedance spectroscopy (EIS) [34,35,36,37,38]. Very recently we published a model used for this purpose that can effectively simulate and predict the behavior of the impedance at low frequencies, which concerns diffusive processes [39]. What was not discussed in that specific study was the interpretation of the high frequency arc that is often discussed as either anode charge transfer [38,40,41,42] or catalyst layer proton transport resistance [43]. The ground-breaking study by M. Heinzman et al. [43] using the distribution of relaxation time analysis (DRT) provides very strong evidence that the high frequency arc adjacent to the *x*-axis intersect (or high-frequency resistance) is in the most common operating conditions of high hydrogen partial pressures dominated by catalyst layer proton transport resistance with subordinate contributions from anode kinetics. In short, they employed a numerical transformation of the EIS data to obtain the frequency-dependent distribution of relaxation times from their recorded impedance spectra. This allows us to easily and precisely deconvolute signals that overlap with other signals to form one semicircle in the Nyquist plot. Controlled modification of operating parameters affecting the electrochemical processes in the MEA (e.g., proton transport, charge transport, or mass transport) was performed. EIS provided evidence that the high-frequency semicircle usually attributed to anode charge transfer is actually dominated by the proton transport in the catalyst layer with a lesser influence of the anode charge transfer under underlying operating conditions. While a lower relative humidity (mainly influencing proton transport) saw a strong increase in the high frequency share of total impedance, the change in hydrogen partial pressure only resulted in a minor change in the high frequency region. At the same time, the increase in oxygen partial pressure strongly increased the medium frequency share of total impedance [43]. Thereby, specific frequency ranges for the different processes were identified. The same technique was used to identify the frequency ranges that are more affected by Cu metal impurities.

The primary goal of the present study was to gain insight into the mechanism of the fast performance decay after break-in procedures and polarization experiments where low voltages are reached without going to potentials above OCP. For that purpose, we compared an in-house-designed de-alloyed and carbon-supported PtCu electrocatalyst with a commercial Pt/Vul benchmark as cathode materials in 25 cm^2^ in-house-fabricated catalyst-coated membranes (CCMs). Additionally, these CCMs were compared to a commercial CCM from Quintech (CCM-H25-N212). The main means of identifying the affected electrochemical processes is the electrochemical impedance spectroscopy and distribution of relaxation time analysis (EIS–DRT). In order to gain complementary insights into the studied phenomena, the thickness of the active catalyst layers and location of Cu before and after single-cell testing was determined by scanning electron microscopy coupled with energy-dispersive X-ray spectroscopy (SEM-EDX) of cryo-cut CCM cross-sections. Ex situ characterization of the catalyst powders was performed using X-ray diffraction (XRD), TF-RDE, and transmission electron microscopy (TEM).

## 2. Materials and Methods

### 2.1. Synthesis of De-Alloyed PtCu3/KB Electrocatalyst

The de-alloyed PtCu_3_/KB electrocatalyst was prepared in four main steps. First, a Cu/KB precursor was synthesized by the modified sol–gel synthesis reported before [44,45,46,47]. In the second step, Pt was deposited on the carbon support by sacrificing a part of Cu using the double passivation galvanic displacement method [22,48]. After the Pt deposition step, the obtained composite was thermally annealed at 800 °C (10 °C min^−1^) for 1 h in an inert atmosphere. After cooling down back to room temperature (RT), the as-prepared PtCu_3_/C electrocatalyst was collected. The Pt loading of 26 wt% and Cu loading of 25 wt% was determined by ICP-OES analysis as described in the Supplementary Information. In the last step, as-prepared PtCu_3_/C electrocatalyst was ex situ chemically activated (ex situ CA) in accordance with the mild activation protocol described already in our previous work [21,49]. Briefly, this includes acid washing of the as-prepared PtCu_3_/C electrocatalyst four times in 1 M acetic acid under carbon monoxide (CO) saturation. The Composition of the PtCu composite was determined by digestion and ICP-OES as described in the Supplementary Information. For more detailed information on material preparation and characterization data, we refer to the previous work of Gatalo et al. [21,49].

### 2.2. XRD Analysis

A PANalytical X’Pert PRO MPD diffractometer (Malvern PANanalytical, Malvern, UK) with Cu Kα1 radiation (λ = 1.5406 Å) was used in order to perform measurements of powder X-ray diffraction (XRD). The measurements were conducted in the 2θ range from 10° to 60° (0.034 step) per 100 s. The X’Celerator detector was fully opened. In order to avoid any overlap of the main peaks, a zero-background Si holder was used.

### 2.3. Scanning Electron Microscopy (SEM) Analysis

Sample preparation for SEM analysis was performed in the same manner as in our previously published work [39]. In short, prior to conducting SEM analysis, the samples were submerged in liquid nitrogen and mechanically cryo-cut using a razor blade. To conduct the SEM analysis, the same field emission scanning electron microscope (ZEISS, Oberkochen, Germany) equipped with an SDD X-MAX 50 Energy-dispersive X-ray (EDX) spectrometer was used.

The cross-sectional images were taken using a standard secondary electron (SE) detector at 1 kV (50 pA). An energy-selective Backscattered detector (EsB) at 1 kV (50 pA) and energy filtering grid set to 400 V were used to acquire the phase-contrast images. In order to determine the position of copper before and after testing, EDX analysis was performed at 7 kV while the probe current was set to 200 pA.

### 2.4. Transmission Electron Microscopy (TEM) Analysis

(S)TEM images were recorded in a probe Cs-corrected scanning transmission electron microscope Jeol ARM 200 CF operated at 80 kV.

### 2.5. Thin-Film Rotating Disc Electrode (TF-RDE) Analysis

Electrochemical evaluation via TF-RDE was conducted in accordance with the procedure described in our previous work [21,22,39] with the same hardware. All electrochemical measurements in this study were conducted in a two-compartment electrochemical cell using a conventional three-electrode system and CompactStat (Ivium Technologies, Eindhoven, The Netherlands) as the potentiostat. A glassy carbon disc electrode (Pine Instruments (Grove City, PA, USA), AFE3T050GC) with a geometric surface area of 0.196 cm^2^ was used as a working electrode, Ag|AgCl as a reference, and a Pt wire as a counter electrode. The potentials are reported against the reversible hydrogen electrode (RHE) for simplicity of comparison, and the transformation is based on measurement of E_RHE_ vs. E_Ag/AgCl_ before and after each RDE experiment. All the measurements were performed using 0.1 M HClO_4_ (Merck, Suprapur, Burlington, MA, USA). Prior to electrode preparation, all the glassy carbon discs were polished with Al_2_O_3_ paste and the glassware was boiled in distilled water. For all catalysts, the thin films were prepared by drop-casting 20 µL of a water-based catalyst ink (1 mg mL^−1^) on the disc electrode. This resulted in complete coverage of the electrode, which was dried under ambient conditions. Prior to mounting the disc electrode on the rotator (Pine Instruments), 5 µL of diluted Nafion solution (ElectroChem (Raynham, MA, USA), 5 wt% aqueous solution, diluted in isopropanol 1:50) was drop-casted on top of the dry electrocatalyst film—once again drying the electrode under ambient conditions.

In the case of Pt/C (Hi-spec 3000 and 4000; Johnson Matthey, London, UK), the catalysts were initially electrochemically activated (EAA) using the protocol that consisted of 200 cycles using the potential window of 0.05–1.2 V_RHE_ (Ar saturated, 300 mV s^−1^, 600 rpm). EAA was followed by an exchange of electrolyte with a fresh one and measurement of the ORR polarization curves in the O_2_-saturated atmosphere (0.05–1.0 V_RHE_, 20 mV s^−1^, 1600 rpm). Prior to the measurement, iR compensation was performed as reported in reference [50]. This was followed by performing CO electrooxidation in Ar-saturated atmosphere (0.05–1.0 V_RHE_, 20 mV s^−1^, no rotation). For ORR, kinetic parameters were determined at 0.9 V_RHE_ by using the Koutecky–Levich equation [51]. The electrochemically active surface area (ECSA_CO_) was calculated from the integral of the CO electrooxidation peak, as described in reference [52]. In contrast to the Pt/C references, the protocol used for the de-alloyed PtCu/KB electrocatalyst consisted of submerging the electrocatalyst in the liquid electrolyte without any potential control in an oxygen-saturated solution. This was followed by both the ORR polarization curve and CO electrooxidation measurements under the same conditions as the Pt/C references. Afterwards, 50 cycles of EAA were performed, followed by an exchange of the electrolyte with a fresh one. Lastly, the ORR polarization curve and CO electrooxidation measurements were repeated. For the de-alloyed PtCu/KB electrocatalyst, ORR and CO electrooxidation data before and after additional EAA cycles were compared.

### 2.6. MEA Fabrication and Electrochemical Characterization

*Electrocatalyst ink preparation*—for this study, three different catalysts were used for ink preparation. Two Pt/C references (Hi-spec 3000 with 20 wt% Pt and Hi-spec 4000 with 40 wt% Pt from Johnson Matthey) and an in-house-synthesized de-alloyed PtCu/KB electrocatalyst (26 wt% Pt and 17 wt% Cu; Figure S1a). In all three cases, the ionomer (5 wt% solution, NS-5 QuinTech) content within the ink was kept constant at 30 wt% of dry electrocatalyst mass (to balance between good proton transport to the active sites in dry conditions and proper water removal in wet conditions [53,54]). This resulted in I/C ratios of 0.8 for Hi-spec 4000, 0.6 for Hi-spec 3000, and 0.8 for the de-alloyed PtCu/KB electrocatalyst. In order to obtain stable inks, a ratio of 0.97:0.03 between 2-propanol (Honeywell, Chromasolv for HPLC, ≥99.9%) and water (Milli-Q) was used for both Pt/C references while the ratio had to be adjusted to 0.85:0.15 for the de-alloyed PtCu/KB. Before preparing the catalyst-coated membranes (CCMs), all catalyst inks were ultrasonicated for 45 min under ice cooling. The list of prepared MEAs can be found in Table 1. Only the CCM marked with * was subjected to a high-humidity stressor 12 h before polarization recording. One commercially available CCM was purchased from QuinTech, Pittsburgh PA, USA (CCM-H25-N212) and used as a reference. All other CCMs were fabricated by using ultrasonic spray coating using an ExactaCoat OP3 from Sono-Tek with the number of deposited layers determining the loading. Previous coating trials with weight monitoring were performed to determine the weight deposited per layer with each ink as described in the Supplementary Information.

CCM fabrication via ultrasonic spray coating (Sono-tek ExactaCoat OP3, Milton, NY, USA) was performed in the same way as in our previous publication [39]. In summary, the membrane was fixed to a porous PTFE filter by vacuum suction, heated to 80 °C, and the catalyst ink was sprayed upon it in a serpentine pattern until target loading was reached. The number of passes needed to reach the loading was determined by the procedure described in the Supplementary Information. A shim mask was used to define the active area of 25 cm^2^. The finished CCMs were left on the PTFE filter plates to dry for ten minutes, and then used for cell assembly.

*Cell assembly and break-in and high humidity stressor*—Assembly and break-in of finished CCMs (Figure S1b) into the testing cell (S^++^ Simulation Services) was performed identically to our previously published procedure [39] and with the same hardware.

In short, the CCMs were placed in between two gas diffusion layers and inserted into the 25 cm² testing cell. (Figure S1c). Reactant gas conditions were set to 100 %RH at 80 °C and 600 mL min^−1^ air/H_2_ at atmospheric pressure. After the cell reached OCV (held for 5 min), cell break-in was performed by switching between 0.4, 0.5, and 0.6 V and holding each point for thirty seconds for a total of three hours. The Potential profile and current density response can be seen in Figure S2b. Only CCM PtCu/KB_0.8* (Table 1) was subjected to a high-humidity stressor directly after the break-in to be later compared to CCM PtCu/KB_0.8, which was subjected to the normal break-in. The stress test was aimed at determining the influence of additional load changes in humid environments, which have been reported to promote Pt dissolution and migration [55,56]. The humidity profile over time compared to normal break-in is depicted in Figure S2a. In total, this leads to 2 h of additional time at 100 %RH, which includes 1 h of additional polarization recording. After the break-in, all cells were held at 0.5 V and the humidification was controlled at 60 %RH overnight (10 h), before polarization curves were recorded using H_2_/air at 80 °C, 250 kPa, 100 %RH, and 600 mL min^−1^.

*Hydrogen crossover-test and in-situ cyclic voltammetry*—Hydrogen crossover and in situ cyclic voltammogramms were recorded in the same manner as previously published [39]. In short, this means supplying the cells with H_2_/N_2_ 500 mL min^−1^ 100% RH until the OCV is stable at 125 mV and then performing a linear potential sweep with 1 mV sec^−1^ from OCV to 0.5 V at differential pressures of 0, 50, and 100 mbar. If the crossover current density was below 15 mA cm^−2^, then the CCMs were used for further testing.

In situ cyclic voltammorgrams with a scan rate of 50 mV s^−1^ at 0 mbar differential pressure were recorded between 70 and 600 mV for three cycles.

*Single-cell electrochemical testing*—All polarization curves were recorded in concordance with our previously published procedures [39], with operating conditions of H_2_/air at 80 °C, 250 kPa, 60 %RH and 600 mL min^−1^ constant flow to maintain an air stoichiometry of 1.1 at the highest current density recorded. Underlying conditions assure that the influence of anode limitations can be neglected, while mass transport limitations on the cathode side appear more distinctly. Before recording polarization, OCV was held for 5 min. Electrochemical Impedance Spectra (EIS) were recorded from 60 kHz to 0.1 Hz with an amplitude of 0.1 A. The operating points of 0.2, 0.3, and 0.4 A cm^−2^ were held for at least five minutes before recording the corresponding spectrum to ensure steady-state conditions. On CCM Pt/Vul_0.6, additional EIS were recorded at atmospheric pressure, 60 %RH, and 100 %RH after finishing the standard testing to verify the influence of the change in the high frequency impedance arc. An overview of the operating conditions is shown in Table 2.

### 2.7. Calculations and Simulation

**Figure 1:**
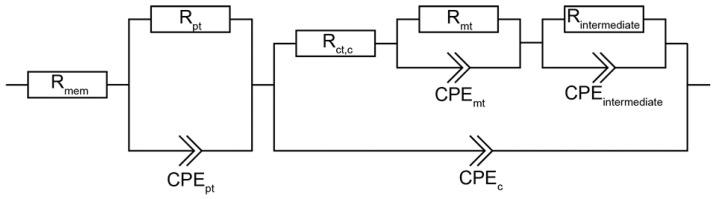
Equivalent circuit model used to determine proton transport (*R*_pt_) and mass transport (*R*_mt_) resistances in the catalyst layers. Reproduced with minor changes from our previous publication [39] under CC by 4.0.

Our recently published equivalent circuit model [39] depicted in Figure 1 was used to determine the resistance contributions of the MEA. We previously showed that this model is able to correctly simulate and interpret low frequency signals. However, as was shown by Heinzmann et al., the ohmic resistance of the high frequency arc (*f_c_* = 3000 − 600 Hz) is dominated by catalyst layer proton transport resistance at the operating conditions used for our experiments [43], with only minor contributions by the anode charge transfer. For this reason we calculated the distribution of relaxation times (DRT) according to referenced methods [43,57], and used the considerations provided by Heinzmann et al. to assign the peaks to electrode processes. It must be noted that only the assignment of the high frequency arc changed, as all other processes were already correctly identified. Additionally, we provide measurements that strongly support Heinzmann et al.’s considerations: The DRT and calculated resistance contributions presented in Figures S12 and S13 show that the value for the high frequency resistance is strongly influenced by relative humidity and only marginally affected by fluid pressure and gas stoichiometry changes. Since an interpretation of anode charge transfer resistance would predict a strong effect from changing hydrogen pressure [58], its interpretation as proton transport resistance better explains the measured effects. Hence, the resistance contribution in the high frequency region calculated using our model was denoted as “proton transport resistance” (*R*_pt_). It should be pointed out that the subordinate anodic contributions cannot be completely forgotten. They can however be neglected at conditions with high hydrogen partial pressure as, e.g., during our measurements 250 kPa, 60 %RH, and high flowrates.

The simulation was performed in the same manner as described in our previously published work [39]. To obtain first estimates of *R*_mt_ and *R*_pt_, the characteristic frequency *f_c_* at which the semicircle or feature appears was used together with estimates of the resistance extracted by placing a semicircle in the feature and reading the value of Z_r_. Equation (2) was the used to calculate the capacitance estimate for the *CPE*. The latter is characterized by Equation (3) with the parameters *P* and *T* [59,60,61,62] where *P* is the *CPE* exponent and *T* is the *CPE* coefficient.

(2) $$f_{c}=\frac{1}{2*\pi *C*R}\;\to C=R*f_{c}*2*\pi$$

(3) $$C_{CPE}=\frac{{\left(T*R\right)}^{\frac{1}{P}}}{R}=T^{\frac{1}{P}}*R^{\left(\frac{1}{P}-1\right)}=T^{P}*R^{\frac{1-P}{P}}$$

The curves were then fitted with an initial value of *P* as one. After this, the characteristic frequency was checked again. The values for *R* were not accepted as correct if the requirements described in our previous work where not met [39]. Numerical correctness of the model was checked by comparing the relative residuals of simulated impedance values (Z_r-sim_, Z_i-sim_) to the experimentally recorded spectra (Z_r_, Z_i_).

## 3. Results and Discussion

**Figure 2:**
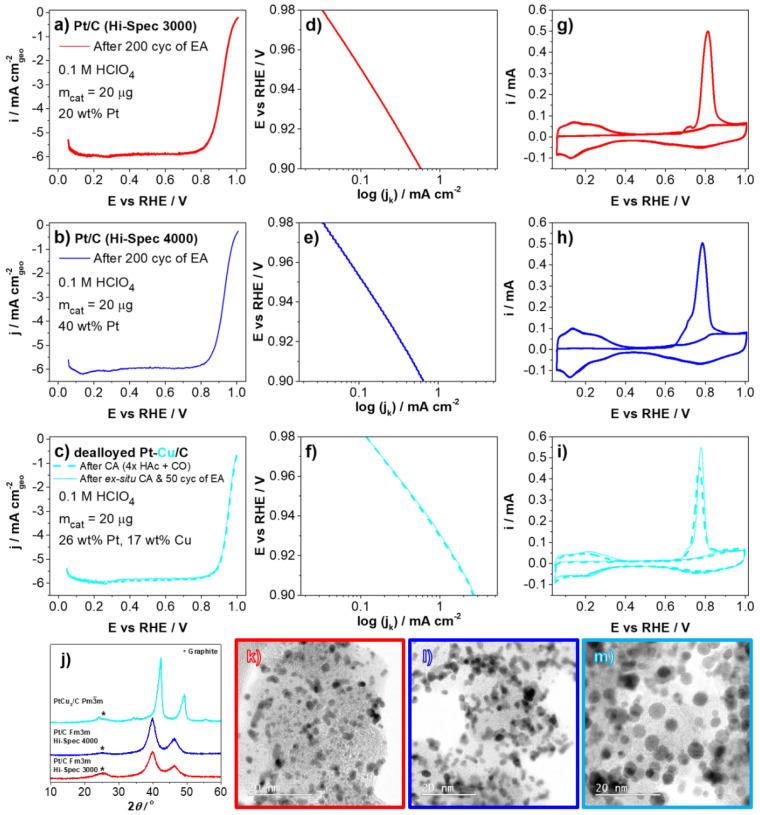
(**a–c**) ORR polarization curves (IR compensated, 1600 rpm, O_2_-saturated) of the catalyst used to manufacture the CCM as stated in Table 1. (**d**–**f**) Calculated Tafel plots and (**g**–**i**) CO “stripping” CVs of Hi-spec 3000, Hi-spec 4000, and proprietary de-alloyed PtCu/KB electrocatalyst measured in half-cell TF-RDE. Relevant electrochemical data based on these measurements are collected in Table S1. (**j**) Comparison of XRD spectra and (**k**–**m**) STEM-BF images of Hi-spec 3000, Hi-spec 4000, and proprietary de-alloyed PtCu/KB. Additional STEM imaging of all catalysts are available in Figures S3–S5. Colors used in this image for graphs as well as image borders are kept constant (Hi-spec 3000 = red, Hi-spec 4000 = blue and PtCu/KB = teal) to more easily distinguish presented data. Reproduced with adaptations from [39] under CC by 4.0. with new results from PtCu/KB.

The results of the ex situ characterization of all used catalysts are depicted in Figure 2. Figure 2a–i show a comparison of TF-RDE results (ORR polarization curves, Tafel plots and CO stripping experiments) for both Pt/C references (Hi-spec 3000 and 4000) and the de-alloyed PtCu/KB electrocatalyst. In accordance with our previous publications [21,22], the TF-RDE results confirmed a much higher ORR activity for de-alloyed PtCu/KB electrocatalyst compared to both Pt/C references. In addition, the ECSA_CO_ value of the PtCu/KB is comparable to that of the Pt/C (Hi-spec 4000) with a similar metal loading. Figure 2j shows the XRD spectra, while Figure 2k–m display a comparison of scanning transmission electron microscopy bright field (STEM-BF) images of all three catalysts at similar magnifications. Both the XRD (Figure 2j) as well as the STEM-BF images (Figure 2k–m) show that crystallite size increases in the following order: Hi-spec 3000 < Hi-spec 4000 < de-alloyed PtCu/KB. The particle sizes measured for PtCu/KB are within the particle size distribution reported by Pavko and Gatalo et al. on the same catalyst material [63]. The alloying effect for de-alloyed PtCu/KB is nicely visible by the shift of 111 and 100 peaks towards higher 2θ angles in contrast to both Pt/C references, as well as by the presence of additional peaks that correspond to the Pm$\bar{3}$m-ordered superlattice structure (Figure 2j).

**Figure 3:**
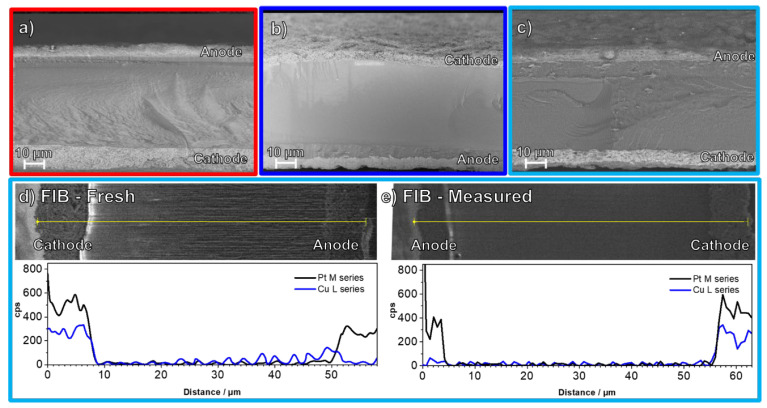
Cryo-cut cross-sections using scanning electron microscopy (SEM) of 25 cm^2^ CCMs with (**a**) CCM Pt/Vul_0.6, (**b**) CCM Pt/Vul_0.8 and (**c**) de-alloyed CCM PtCu/KB_0.8. The Hi-spec 3000 electrocatalyst was used on the anode for all samples. More SEM analysis is available in Figures S6–S8, while the thicknesses are collected in Table S2. Additional comparison of cryo-cut cross-sections and EDX line-scans of CCM PtCu/KB_0.8 after FIB polishing (**d**) fresh and € measured CCM. Semi-quantitative EDX analysis of both CCMs measured at different areas of the cryo-cut and FIB-polished cross-sections is available in Figure S10.

Figure 3 shows cryo-cut cross-sections using scanning electron microscopy (SEM) of 25 cm^2^ CCMs. The Pt/C 20 wt% (Hi-spec 3000; 11.7 ± 0.3 µm) is roughly twice as thick as the Pt/C 40 wt% (Hi-spec 4000; 4.4 ± 0.3 µm) reference (Figure 3a,b). The thickness of the cathode with de-alloyed PtCu/KB is in between those of both Pt/C references (8.4 ± 0.2 µm) (Figure 3c). In all the cases, the thickness of the anode layer was kept constant within the experimental error (4.4 ± 0.2 µm across one anode active layer and 4.4 ± 0.3 µm across all three different anode active layers). 

Furthermore, although the thickness of a commercial Quintech CCM cathode is comparable to the other cathodes (6.8 ± 1.8 µm, see also Supporting Information, Figure S9 for SEM cross-section), it needs to be stressed that in this case, the loading is considerably higher, which has to be taken into account when analyzing the performance results.

Finally, we performed EDX line-scan analysis of both fresh (Figure 3d) and measured (Figure 3e) cryo-cut cross-sections of CCMs with de-alloyed PtCu/KB electrocatalyst on the cathode and Hi-spec 3000 electrocatalyst on the anode after focused ion beam (FIB) polishing. This analysis was performed to exclude any possible Cu migration from the cathode to the membrane or even the anode over the course of the single-cell measurement. Such a phenomenon was observed during the work of Yu et al. [25] after degradation and could, thus, be relevant for correct interpretation of the polarization curve and EIS measurements.

Additionally to EDX line-scan measurements, semi-quantitative EDX analysis was carried out on both CCMs (see Supporting Information, Figure S10a,b), which definitely confirmed absence of Cu outside the cathode for both fresh and measured CCMs. In the case of the fresh CCM (Figure 3d), an artifact signal for Cu was visible between ~40–50 µm (near the anode) due to electron beam damage of the Nafion membrane.

**Figure 4:**
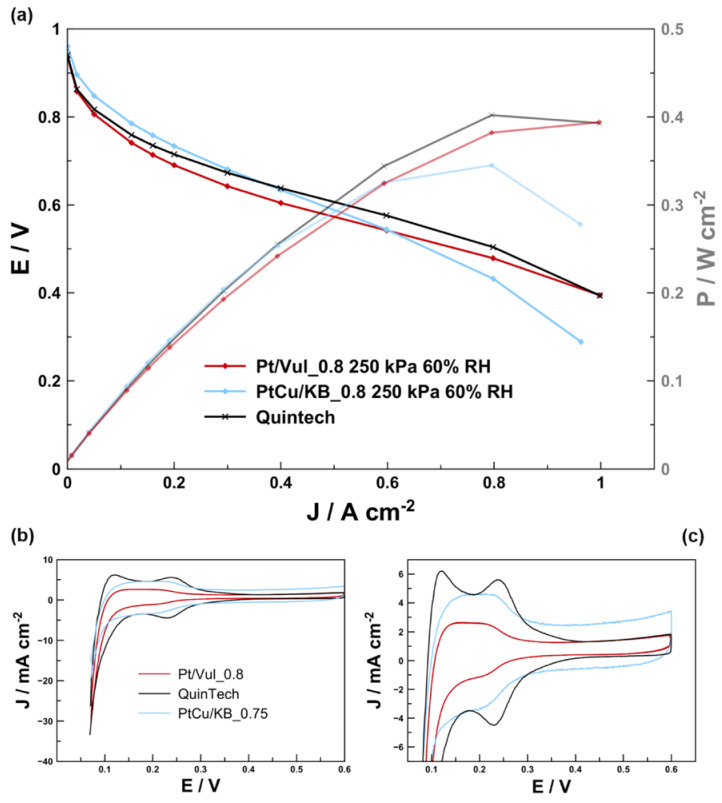
Comparison of best-performing polarization curves (**a**) and in situ cyclic voltammograms of Pt/Vul_0.8, PtCu/KB_0.8 and the Quintech CCMs (**b**). The CV of low-loading CCMs are additionally represented at a smaller axis scaling (**c**) to better recognize the small HUPD peak. The polarization curves were recorded under air/H_2_ (600 mL min^−1^), 250 kPa, T_C_ = T_A_ = 80 °C, 60 %RH. CVs were recorded at N_2_/H_2_ (600 mL min^−1^), 100 kPa, T_C_ = T_A_ = 80 °C, 100 %RH.

Figure 4a shows a comparison of 25 cm^2^ single-cell polarization curves of in-house-fabricated CCMs as well as the commercial QuinTech CCM directly after the break-in procedure. Lower power density was observed compared to Ramaswamy et al. and Gatalo et al. [6,7,8], yet a higher power density was observed than Falina et al. [26]. This was attributed to differences in flow field/gasket/GDL combinations and consequent compression of the GDL [64]. The overall performance expressed in power density is impacted by the material composition, manufacturing history, single cell hardware, and reactant stoichiometry and varies widely over the relevant literature [3]. The experimental conditions in the present study were designed to study the fast performance decay after break-in by material comparison, and the limiting currents were high enough to reach this goal as observed by the fast onset of current density degradation in Figure S2b. No further flow field/gasket/GDL optimization to reach higher performance was undertaken.

At low current densities, the CCM with de-alloyed PtCu/KB electrocatalyst clearly outperformed both CCMs with Pt/Vul. In addition, despite the considerably lower Pt loading (almost five times), the CCM with the de-alloyed PtCu/KB electrocatalyst also outperformed the Quintech CCM at current densities up to approx. 0.4 A cm^−2^. We presume that this is a result of both better utilization of Pt atoms (higher ECSA) as well as higher intrinsic activity. The ECSA calculated from the integral of the hydrogen desorption peak seen in Figure 4b,c is listed in Table 3 and is increasing in the order Quintech ≈ Pt/Vul_0.8 < PtCu/KB. In any case, the present results demonstrate that the improved kinetic ORR activities for PtCu alloy catalysts observed in TF-RDE can be translated to the single-cell level and that the performance decay must be for a reason other than kinetic limitations. The ECSA values of PtCu/KB (57.9 m² g^−1^) are close to the 68 m² g^−1^ reported by Yu et al. [25]. These small differences can stem from lower wt% of metal on HSC, 34% in the case of Yu et al. [25], while we used 43% and different Cu to Pt ratios. In addition, it can be seen that upon using the same cell hardware and operating conditions, the CCM with de-alloyed PtCu/KB electrocatalyst exhibited higher performance at high potentials (~0.6–0.95 V) with a reasonably high efficiency [8,65,66,67,68,69] compared to both CCMs with commercial Pt/Vul catalysts and the commercial QuinTech CCM. However, at the current densities of ≥0.6 A cm^−2^, the CCM with de-alloyed PtCu/KB electrocatalyst suffers from rapidly increasing polarization. In CCMs with Pt/Vul references, such limitations are less severe and become only apparent at current densities higher than approx. 0.8 A cm^−2^. Compared to the CCMs that use Pt/Vul in Figure 4a, the voltage of CCM with de-alloyed PtCu/KB electrocatalyst drops quickly above ~0.6 A cm^−2^, indicating diffusion-related issues. Thus, according to the previous findings, this issue can in part be initially attributed to the thicker active layer (~8–9 µm) [39]. An additional reason, however, could also be related to the type of carbon support (Ketjen Black EC300J) used to prepare the de-alloyed PtCu/KB electrocatalyst. It has been previously shown by Yarlagadda et al., Padgett et al. and Ramaswamy et al. that Ketjen Black EC300J exhibits a highly microporous structure that is on the one hand beneficial for the activity, but on the other hand detrimental for the accessibility of reactant gases to the active particles inside the micropores [8,68,69,70]. Thus, the accessibility of active nanoparticles could once again be responsible for diffusion-related limitations. Furthermore, as presented in Figure 4b), the CV of PtCu supported on Ketjen Black EC300J exhibits a very large capacitive current owing to the almost 3.5× higher BET surface area in contrast to Vulcan XC72 [68]. The presence of such a large capacitive current indicates a large carbon surface area and confirms that acetic acid did not alter the carbon structure significantly, and that the catalyst contains a very large high microporous volume fraction, which could have reduced the accessibility of the Pt nanoparticles. By close inspection of the current density response over the duration of the break-in performed 10 h before all polarization measurements (shown in Figure S2b of the Supplementary Information), it is noticed that, for both PtCu/KB CCMs, performance decay already starts after ~1 h of load cycling at 100 %RH, while CCM Pt/Vul_0.8 showed a stable current density response. While the previous paragraph discussed the diffusion-related possibilities for the steeper voltage decrease of the CCM containing the de-alloyed PtCu/KB, the linear region of the polarization curve is, in fact, typically dominated by the ohmic voltage losses from the ionomer electrolyte resistance.

**Figure 5:**
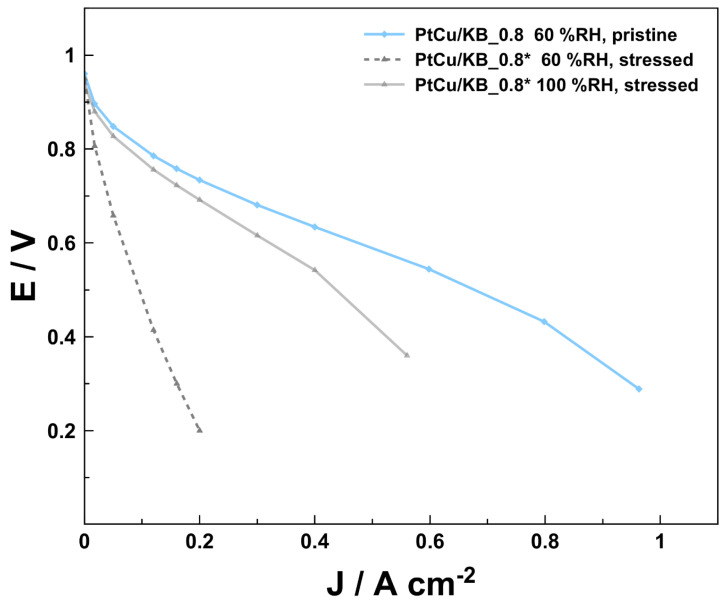
Effect of high-humidity stressor 12 h before performance test at 60 %RH and 100 %RH. All polarization curves were recorded at 80 °C, 250 kPa H_2_/air, 600 mL min^−1^ fixed flow. The polarization profile of the stressor is shown in Figure S14 of the Supplementary Information.

Following the conclusions of previous reports [24,25,26], the main effect on the performance loss of the CCM containing the de-alloyed PtCu/KB could also be the effect of Cu damaging the ionomer electrolyte. Since, according to the SEM-EDX results in Figure 3, Cu is only present in the cathode catalyst layer, the damage to the ionomer electrolyte very likely occurred at the cathode, but more specifically, close to the active sites (at the nm scale). To better understand the role of liquid water combined with low potentials, an additional single-cell polarization curve was measured with a new CCM PtCu/KB_0.8* (hereinafter referred to as stressed CCM) that was manufactured identically to the CCM PtCu/KB_0.8 presented in Figure 4 (hereinafter referred to as pristine CCM PtCu/KB_0.8). However, in the case of CCM PtCu/KB_0.8*, the CCM was subjected to a high-humidity stressor after the break-in and 10 h before the polarization curve measurement at 60% RH (see profile in Figure S2a). The polarization curve measurements of both pristine CCM PtCu/KB_0.8 and stressed CCM PtCu/KB_0.8* are compared in Figure 5. Due to the performance peak and subsequent drop in performance in the break-in current densities shown in Figure S2b, we assume that, already during the cell break-in, a small amount of fresh Cu was dissolved and negatively affected the performance of both pristine CCM PtCu/KB_0.8 and stressed CCM PtCu/KB_0.8*. This indicates that a very high degree of care must be taken when handling Cu-containing CCMs, and strategies to avoid Cu dissolution should be developed to deploy the full potential of the highly active PtCu catalyst system. The drastic performance decay after exposure to the humidity stressor indicates that the stressed CCM PtCu/KB_0.8*, the twice-aslong exposure to high humidity, and low cell voltages may accelerate the performance decay. CCM PtCu/KB_0.8* indicates typical signs of very high electrolyte resistance and mass transport limitations. Due to the poor performance of the stressed CCM PtCu/KB_0.8*, no stable operating point for EIS evaluation could be found. Additionally, while the performance of the stressed CCM PtCu/KB_0.8* did improve upon increasing the humidity from 60 to 100 %RH, it still did not reach the performance of the pristine CCM PtCu/KB_0.8. The most probable cause for the significantly higher performance decay of the stressed CCM PtCu/KB_0.8* is the much higher amount of dissolved Cu ions that resulted in significant damage to the ionomer electrolyte directly at the catalyst/ionomer interface. The origin of the dissolved Cu ions is most likely a result of the prolonged time at 100 %RH at high current densities during the high-humidity stressor, which have previously been reported to accelerate Pt dissolution [6,55,56]. Thus, in the case of the de-alloyed PtCu/KB electrocatalyst, this might have damaged the Pt overlayer and exposed the Cu-rich core to the highly acidic ionomer electrolyte. The freshly dissolved Cu from the core then promoted the degradation of the ionomer directly at the catalyst/ionomer interface. This indicates that it is crucial to properly pre-treat Cu-containing catalysts and CCMs by chemical and electrochemical means, to use properly high ionomer content, and to avoid high humid conditions. An additional origin of the Cu ions could also be as a result of left-over Cu impurities previously embedded in the carbon matrix after the electrocatalyst de-alloying. This is because it has been previously shown that also carbon corrosion is promoted by high-humidity conditions [71], which could have also freed up the previously embedded Cu ions and dissolve them. The results observed in the case of stressed CCM PtCu/KB_0.8* (Figure 5) indicate that in the case of pristine CCM PtCu/KB_0.8 (Figure 4) in addition to the diffusion-related possibilities (use of microporous carbon support and cathode thickness), Cu ions might also play a role in steeper decrease in the voltage in comparison to the pure Pt CCMs.

**Figure 6:**
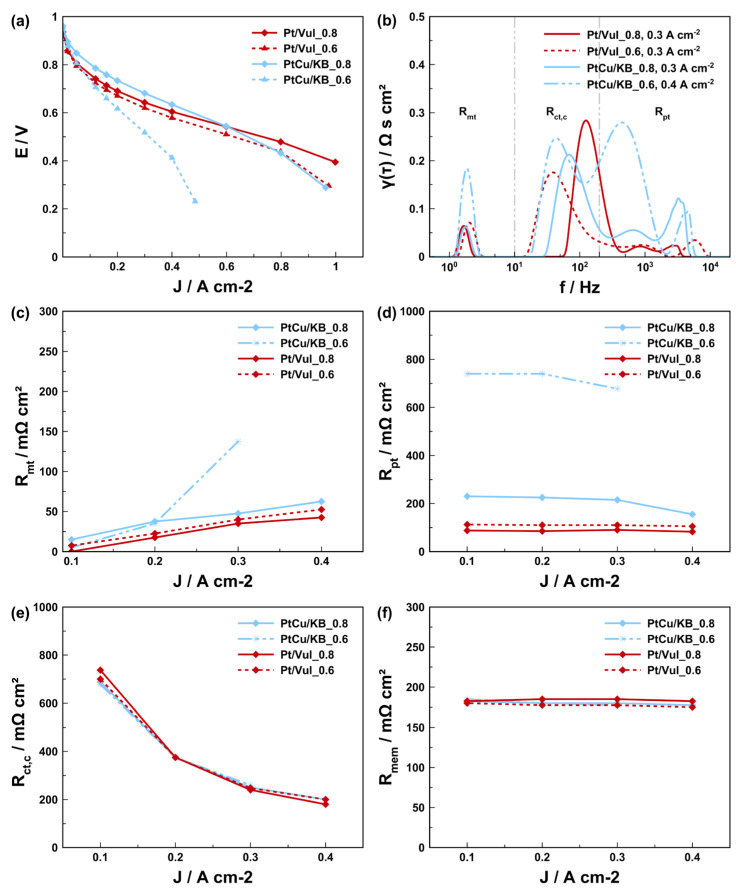
Polarization curves at 80 °C, 250 kPa H_2_/air, 600 mL min^−1^ of prepared CCMs with variation of I/C and catalyst material. (**a**) Comparison of DRT analysis of recorded EIS at 0.3 A cm^−2^ (**b**). Comparison of the resistance contributions determined by the simulation. Trend of mass transport resistance (*R*_mt_), (**c**) proton transport resistance (*R*_pt_), (**d**) cathode charge transfer resistance, (**e**) and membrane resistance (**f**) with increasing current densities. Comparison of simulated and measured impedance spectra and DRT analysis results can be found in Figures S11–S13 of the Supplementary Information together with relative residuals.

In order to better quantify and understand the performance decay and understand its impact on the electrochemical processes in the MEA, we carried out a comparative EIS study of all the evaluated in-house-fabricated CCMs (Table 1). In addition to CCMs with I/C of 0.8, this also includes the CCMs with I/C of 0.6 for both the CCMs with Pt/Vul as well as the CCMs with the de-alloyed PtCu/KB electrocatalyst. The equivalent circuit model presented in Figure 1 was used to quantify the contributions of single voltage loss mechanisms on the overall voltage loss [39]. Polarization curves as well as the recorded EIS spectra and the corresponding resistance contributions calculated from the model simulation are presented in Figure 6. A comparison of the simulated and experimental impedance spectra and the corresponding residual analysis are presented in Figure S11 of the Supplementary Information. In these measurements, we mostly focused on the current densities corresponding to the linear region of the polarization curves. The spectra show that the membrane resistance is within the same order of magnitude for all the measured CCMs. Thus, in the case of all CCMs with the de-alloyed PtCu/KB electrocatalyst, potential contributions to the resistance due to Cu migration from the cathode to the membrane or the anode can be excluded based on the results from the EDX analysis of both fresh (Figure 3d) and measured (Figure 3e) FIB-polished cryo-cut cross-sections. The most distinctive difference in the EIS spectra in Figure S11 is for the CCMs with de-alloyed PtCu/KB electrocatalyst; three distinct time constants (τ_1_, τ_2_ and τ_3_) are clearly visible. On the other hand, on both CCMs with Pt/Vul the τ_1_ arc is almost indistinguishable from τ_2_, creating a 45° slope in the region where τ_1_ appeared for de-alloyed PtCu/KB. DRT analysis following the theoretical basis presented and considerations explained in the modelling section of previous work by Heinzman et al. [43] attributes the arc τ_1_ to the proton transport resistance (*R*_pt_). Results of the DRT analysis at varying operating conditions of the present study presented in Figure S12 further solidify the view that τ_1_ is connected to *R*_pt_, with only subordinate and negligible anode contributions at the underlying operating conditions. While the change in I/C ratio slightly affected Pt/Vul-based CCMs performance, it also considerably affected polarization behavior of the CCMs with the de-alloyed PtCu/KB electrocatalyst (Figure 6a). The CCM PtCu/KB_0.6 indicates signs of higher ohmic resistance and very early onset of diffusion limitations. This is consistent with the results provided by the EIS measurements and calculated resistance trends (Figure 6b–f). Comparison of DRT analysis results of the EIS recorded at 0.3 A cm^−2^ (Figure 6b) indicates a large increase in the peak area in the frequency range indicative for *R*_pt_ marked by the grey lines. At the same time, the peak area trend of charge transfer resistance (*R*_ct_) follows the activity trend of the respective catalyst determined by TF-RDE, as presented in Table S1 of the Supplementary Information. The trend of mass transport resistance (*R*_mt_) with increasing current density, calculated from the EC model when matching it to the experimental EIS spectra in Figure S11, is shown in Figure 6c. The trend is very similar for the CCM PtCu/KB_0.8 and the two pure Pt CCMs Pt/Vul_0.8 and Pt/Vul_0.6. It is observed that the mass transport resistance of Pt/Vul_0.6 is slightly higher than that of Pt/Vul_0.8, which is explained by the thicker catalyst layer (12 µm vs. 5 µm) of CCM Pt/Vul_0.6 [39]. At the same time, the *R*_mt_ of CCM PtCu/KB_0.8 with an 8 µm catalyst layer is considerably higher than *R*_mt_ of CCMs Pt/Vul_0.6 and Pt/Vul_0.8. This effect is caused by the microporous volume of the Ketjen Black EC300J carbon, which leads to a large number of inaccessible active nanoparticles and hence mass transport limitations by depletion of free active sites at high current densities [68,69,70,72]. This effect is exacerbated when the I/C ratio is lowered, since the ionomer contact area with the metal particles is lower, which leads to a much quicker depletion of free active sites. Therefore, even though the *R*_mt_ of CCM PtCu/KB_0.6 is initially lower then the *R*_mt_ of CCM PtCu/KB_0.8 at low current densities, it quickly increases with higher currents and is already higher by a factor of ~3 at 0.3 A cm^−2^. Since at 0.4 A cm^−2^ the polarization behavior of CCM PtCu/KB_0.6 is no longer linear, no EIS spectra above 0.3 A cm^−2^ have been measured. Trends for *R*_pt_ are shown in Figure 6d and exhibit almost constant values with increasing current density, reflecting their ohmic behavior. A small linear decrease with increasing current density is attributed to higher amounts of product water and hence better hydration. Charge transfer resistance in Figure 6e only differs significantly at 0.1 A cm^−2^ and fits the activity trend measured ex situ presented in Figure 2 (R_Ct,C_: Pt/Vul 40% > Pt/Vul 20% > PtCu/KB), whereas the membrane resistance in Figure 6f did not change significantly between the CCMs. It is also shown that catalyst layers prepared with PtCu/KB exhibit a higher resistance contribution from proton transport in the catalyst layer (*R*_pt_). Furthermore, the reduction in the I/C ratio affected the *R*_pt_ of the PtCu/KB CCMs to a much larger extent than it did for the *R*_pt_ of Pt/Vul-based CCMs. While the reduction in the I/C ratio from 0.8 to 0.6 increased the average *R*_pt_ by ~24 Ω cm² for Pt/Vul-based CCMs, it increased it by ~536 Ω cm² for PtCu/KB-based CCMs.

## 4. Conclusions

In the present work, a de-alloyed PtCu/KB electrocatalyst was studied in a single-cell environment and compared with in-house-fabricated CCMs containing commercial Pt/Vul catalysts. On the one hand, mass transport problems were clearly observed at the higher current densities ≥0.8 A cm^−2^. This is partly due to the pore structure of Ketjen Black EC 300J and partly due to the relatively thick (approx. 7 µm versus 5 µm) catalyst layer. This is mainly influenced by the type of carbon support with no observable change in the timescale of measurements. While the detrimental effect of Cu alone was already observed previously, it has been debated whether it would disrupt proton transport in the membrane or migrate to the anode and affect HOR kinetics, or interfere with ORR kinetics on the cathode by Cu accumulation on the catalyst surface.

Our research revealed that the performance decay sets in during cell break-in for all PtCu-based CCMs. The investigations using SEM-EDX showed that the performance decay occurred without Cu migrating out of the cathode catalyst layer. The analysis of resistance contributions using EIS–DRT showed that the proton transport resistance (*R*_pt_) in the cathode catalyst layer is twice as high for PtCu-catalyzed CCMs compared to Pt-catalyzed CCMs. Reducing the ionomer to carbon ratio (I/C) from 0.8 to 0.6 increased the *R*_pt_ by a factor of ~3.3, while it increased only by a factor ~1.2 for Pt-catalyzed CCMs. The main reason for the rapid drop in performance is a disruption of proton transport in the cathode catalyst layer, presumably due to damage to the ionomer as a result of Cu ion contamination. It was additionally found that the performance degradation is accelerated by prolonged operation at relative humidity above 60%.

Despite these challenges, we can make the following considerations to translate the superior PtCu performances often observed in TF-RDE to the cell level:

(i) Proper chemical activation process for the PtCu alloy system that effectively removes Cu impurities from both the Pt alloy nanoparticle surface and the carbon matrix. The resulting platinum overlayer should be thick enough to present a kinetic barrier for Cu migration from the core to the surface.
(ii) Completely avoid or limit operation of the cell at relative humidity above 60 %RH during all phases of operation to avoid additional dissolution of fresh Cu and consequent damage to the ionomer electrolyte near the catalyst/ionomer interface.
(iii) Develop cathode ionomers resistant to Cu ions or metal ion contaminations more generally.

## Figures and Tables

**Table 1:** List of prepared CCMs. The CCM marked with * was subjected to a high-humidity (100 %RH) stressor 12 h before testing. All self-made anode catalyst layers were 4.4 ± 0.3 µm thick with an I/C ratio of 0.6.

CCM Code	Cathode Catalyst	Pt-Loading Loading/mg cm^2^	Cathode I/C	Cathode Catalyst Layer Thickness/µm	Anode Catalyst	Pt-Loading Loading/mg cm^2^
Pt/Vul_0.8	Pt/Vul 40% (HiSPec 4000)	0.125	0.8	4.4 ± 0.3	Pt/Vul 20% (HiSPec 3000)	0.05
Pt/Vul_0.6	Pt/Vul 20% (HiSPec 3000)	0.125	0.6	11.7 ± 0.3	Pt/Vul 20% (HiSPec 3000)	0.05
PtCu/KB_0.8	PtCu3/KB 43%	0.125	0.8	8.4 ± 0.2	Pt/Vul 20% (HiSPec 3000)	0.05
PtCu/KB_0.8 *	PtCu3/KB 43%	0.125	0.8	8.4 ± 0.2	Pt/Vul 20% (HiSPec 3000)	0.05
PtCu/KB_0.6	PtCu3/KB 43%	0.125	0.6	n.a.	Pt/Vul 20% (HiSPec 3000)	0.05
QuinTech	Pt/C	0.6	n.a.	n.a.	Pt/C	0.3

**Table 2:** Overview of operating conditions used for polarization and EIS measurements on the CCMs.

CCM Code	Fuel/Oxidant	θ/°C	p/kPa	RH/%	V/mL min^−1^	CCM Code
Pt/Vul_0.8	H_2_/air	80	250	60	600	Pt/Vul_0.8
Pt/Vul_0.6	H_2_/air	80	250	60	600	Pt/Vul_0.6
Pt/Vul_0.6	H_2_/air	80	100	60	600	Pt/Vul_0.6
Pt/Vul_0.6	H_2_/air	80	100	100	600	Pt/Vul_0.6
Pt/Vul_0.6	H_2_/air	80	100	60	800	Pt/Vul_0.6
PtCu/KB_0.8	H_2_/air	80	250	60	600	PtCu/KB_0.8

**Table 3:** ECSA calculated from the in situ CVs presented in Figure 3b,c using the integral of the hydrogen desorption peak.

CCM Code	ECSA/m² g_Pt_^−1^
Pt/Vul_0.8	32.8
Quintech	31.0
PtCu/KB_0.8	57.9

## Supplementary Materials

The following supporting information can be downloaded at: https://www.mdpi.com/article/10.3390/ma16093544/s1.
