# Supplementary material for: Mechanistic Study of Fast Performance Decay of PtCu Alloy-based Catalyst Layers for Polymer Electrolyte Fuel Cells through Electrochemical Impedance Spectroscopy
Source: Materials (Basel). 2023 May 5;16(9):3544. doi: 10.3390/ma16093544 (PMC10180127; doi:10.3390/ma16093544)
Supplement: Supplementary file 1 [file materials-16-03544-s001.zip › materials-2315165-supplementary.pdf]

Supplementary information: *"Mechanistic study of fast performance decay of Pt-Cu alloy based catalyst layers for polymer electrolyte fuel cells through electrochemical impedance spectroscopy"*

Maximilian Grandi 1,* , Matija Gatalo 2 , Ana Rebeka Kamšek 2, Gregor Kapun 2 , Kurt Mayer 1 ,

Francisco Ruiz-Zepeda 2 , Martin Šala 3, Bernhard Marius 1, Marjan Bele 2, Nejc Hodnik 2 , Merit Bodner 1 ,

Miran Gaberšˇcek 2 and Viktor Hacker 1

1 Institute of Chemical Engineering and Environmental Technology, Graz University of Technology,

Inffeldgasse 25/C, 8010 Graz, Austria

2 Department of Materials Chemistry, National Institute of Chemistry, Hajdrihova 19, 1000 Ljubljana, Slovenia

3 Department of Analytical Chemistry, National Institute of Chemistry, Hajdrihova 19, 1000 Ljubljana, Slovenia

* Correspondence: maximilian.grandi@tugraz.at

Digestion and ICP-OES for determination of PtCu/KB composition.

All reagents used were of analytical grade or better. For sample dilution and preparation of standards, ultrapure water (18.2 MΩcm^{-1}, Milli-Q, Millipore) and ultrapure acids (HNO_3 and HCl, Merck-Suprapur) were used. Standards were prepared in-house by dilution of certified, traceable, inductively coupled plasma (ICP)-grade single-element standards (Merck CertiPUR). A Varian 715-ES ICP optical emission spectrometer was used. Prior to ICP-OES analysis, de-alloyed PtCu/KB electrocatalyst was weighted (approximately 10 mg) and digested using a microwave-assisted digestion system (Milestone, Ethos 1) in a solution of 6 mL HCl and 2 mL HNO_3. Samples were then filtered and the filter paper was again submitted to the same digestion protocol. These two times digested samples were cooled to RT and then diluted with 2 %v/v HNO_3 until the concentration was within the desired concentration range.

Electrocatalyst loading determination and CCM preparation

Prior to the preparation of the CCMs we coated a gas diffusion layer (Sigracet 29BC) to determine the number of necessary spraying passes (weight per pass) to reach the desired loading (0.125 mg_{Pt} cm^{-2}) for the cathode/anode. For that, each of the electrocatalyst inks was sprayed 3 times 20 cycles (40 layers) on the GDL. The GDL was weighed before as well as after 20, 40 and 60 spraying cycles. The weighed mass after 20, 40 and 60 spraying cycles was averaged to determine the weight of electrocatalyst per spraying cycle. **Table S2 and S3** show the results along with high precision and repeatability towards catalyst loading achieved with the ink-preparation and coating process.

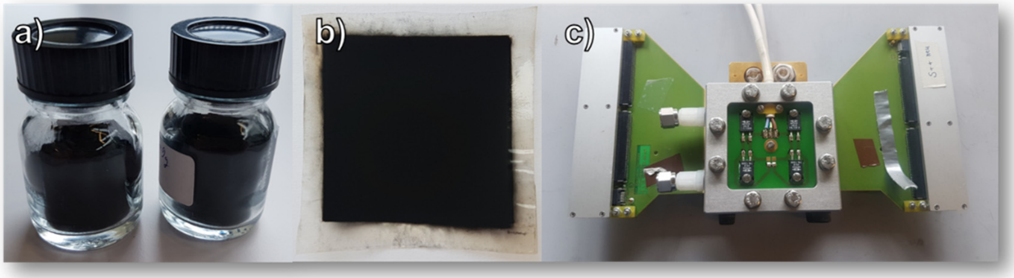

Figure S1: (a) Electrocatalyst powder, **(b)** CCM fabricated with Ultrasonic spray-coater (Sonotech ExactaCoat OP3) and **(c)** assembled single cell.

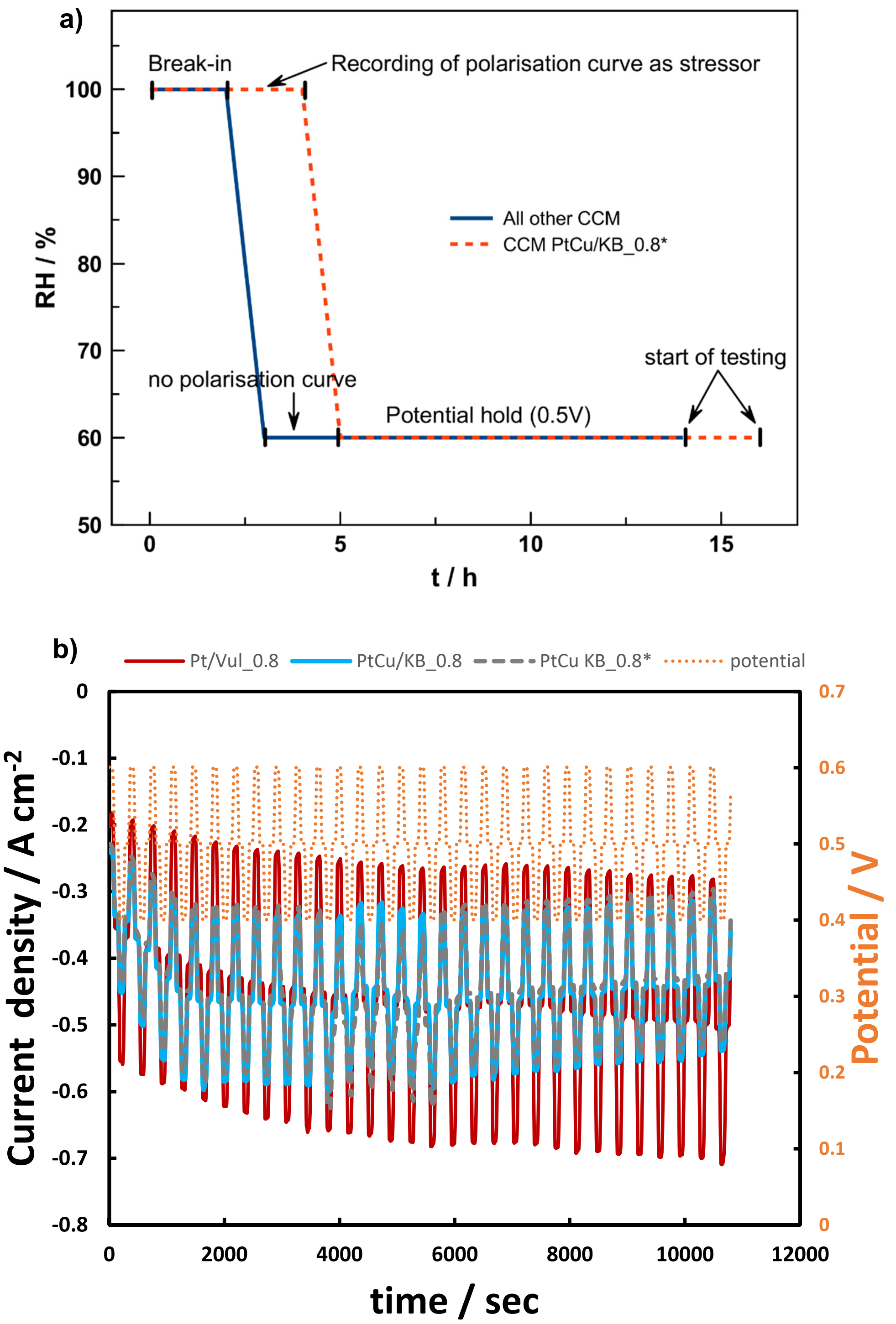

Figure S2: Profile of relative humidity over the time and history of treatment for the CCMs before the start of performance testing and EIS recording **a)**. Potential hold steps and current response of Pt/Vul_0.8, PtCu/KB_0.8 and PtCu/KB_0.8* during break-in **b)**.

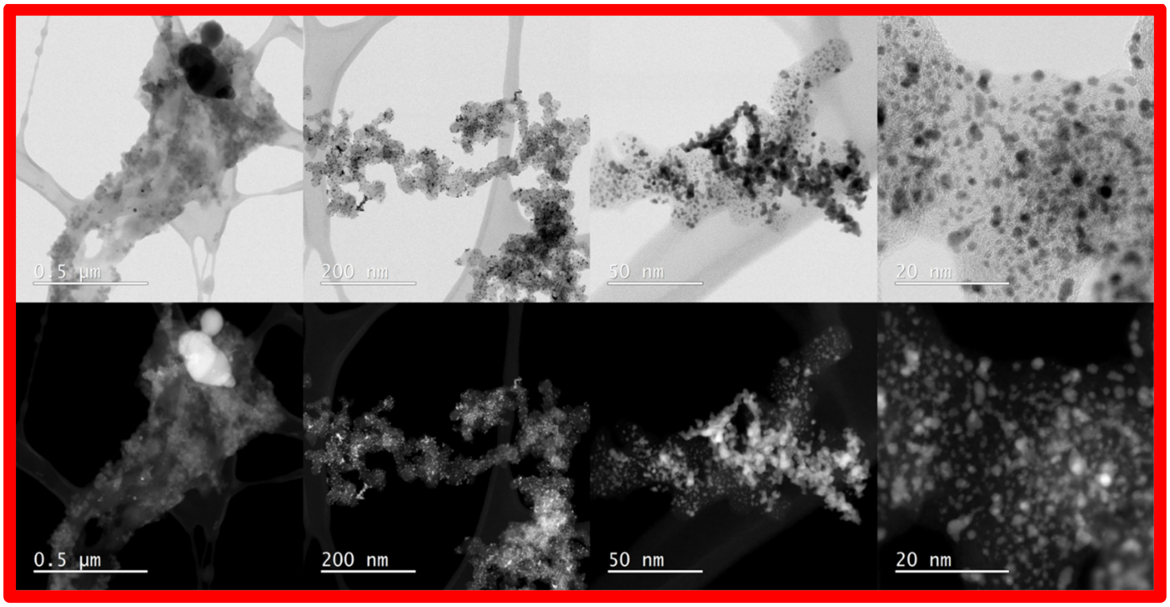

Figure S3: STEM BF and HAADF imaging of Pt/C Hi-spec 3000 at different magnifications.

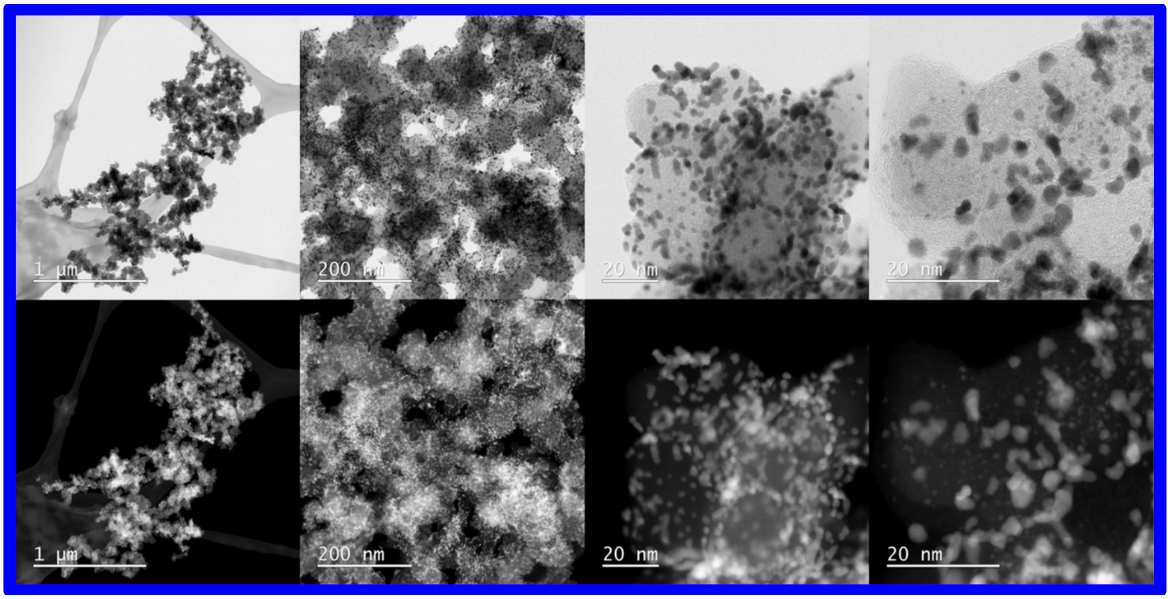

Figure S4: STEM BF and HAADF imaging of Pt/C Hi-spec 4000 at different magnifications.

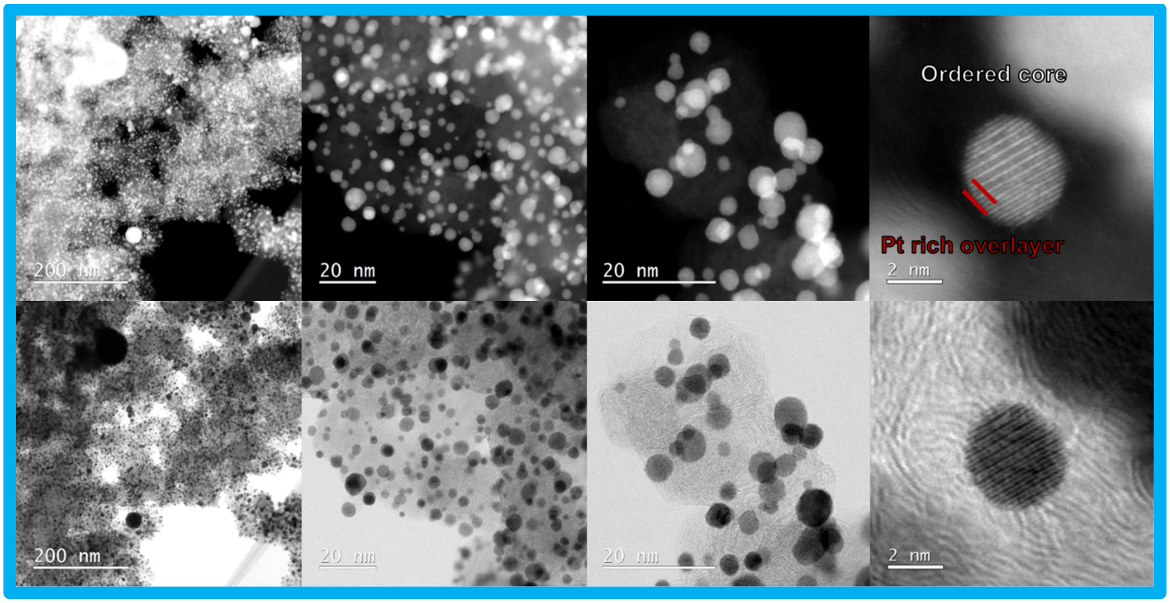

Figure S5: STEM HAADF and BF imaging of dealloyed Pt-Cu/C at different magnifications.

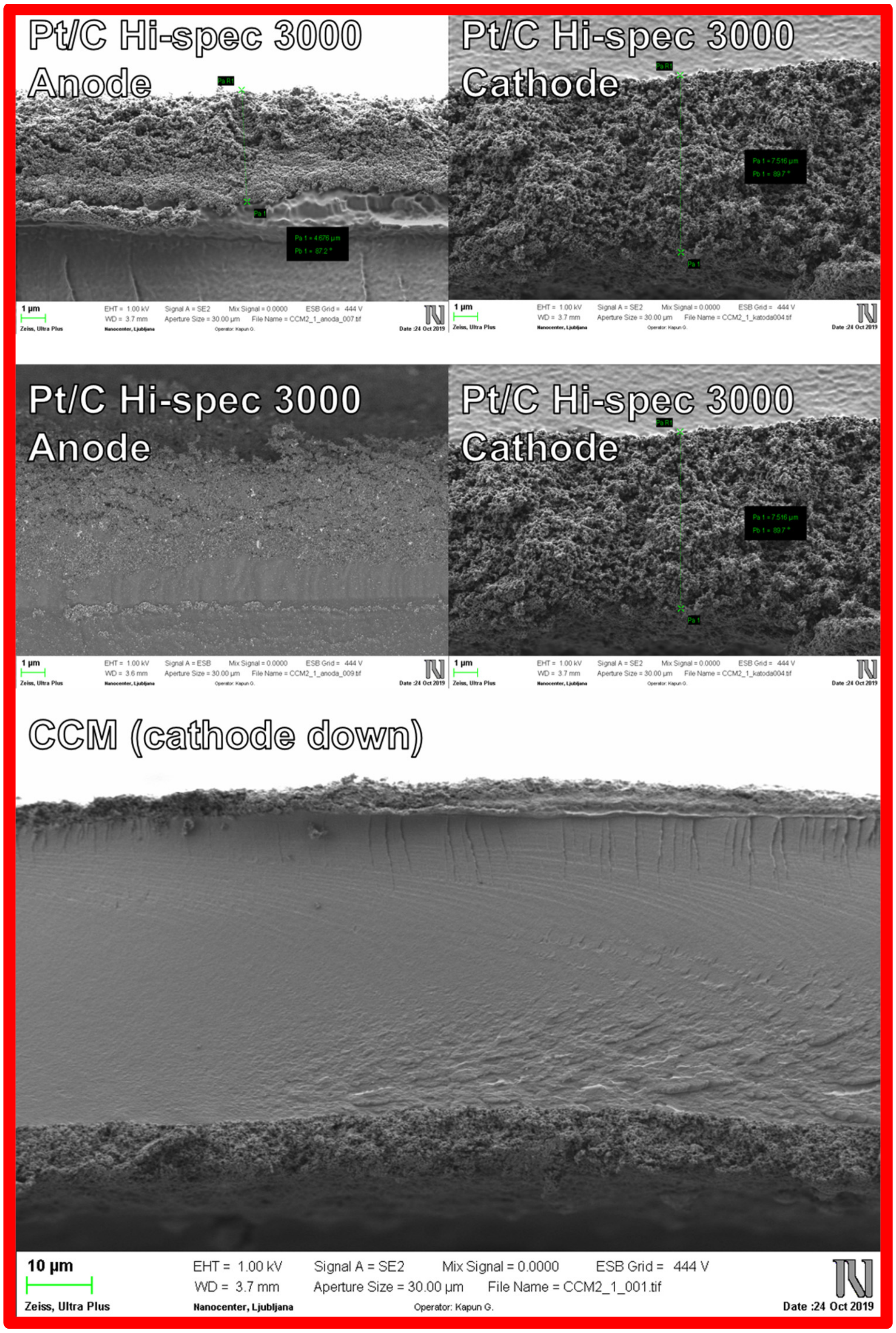

Figure S6: Cross-section SEM analysis of in-house fabricated CCM with 0.125 mg_{Pt} cm^{-2} Pt/C Hi-spec 3000 cathode, 0.05 mg_{Pt} cm^{-2} Pt/C Hi-spec 3000 anode and ~50 µm membrane (NAFION NM 212, DuPont).

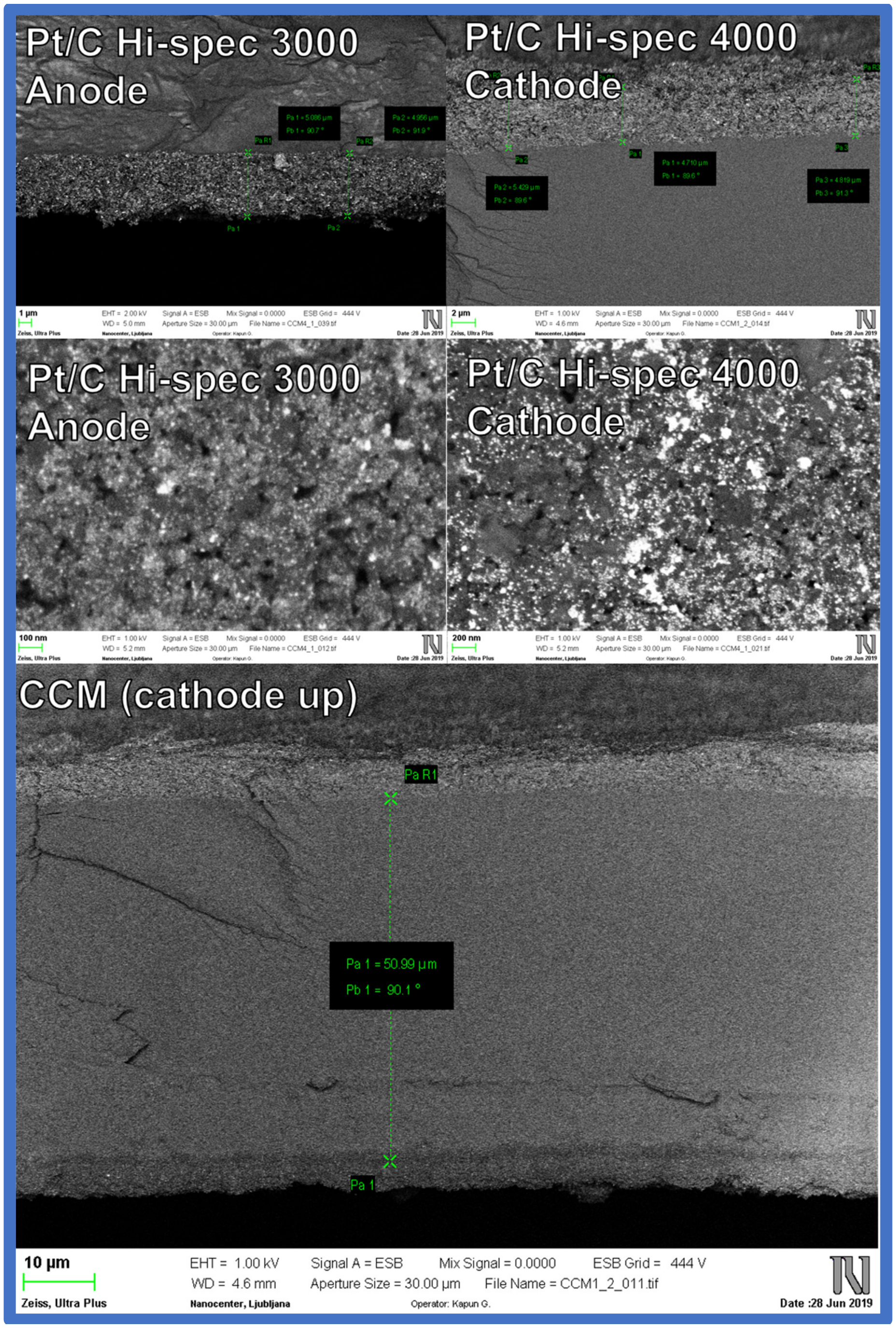

Figure S7: Cross-section SEM analysis of in-house fabricated CCM with 0.125 mg_{Pt} cm^{-2} Pt/C Hi-spec 4000 cathode, 0.05 mg_{Pt} cm^{-2} Pt/C Hi-spec 3000 anode and ~50 µm membrane (NAFION NM 212, DuPont).

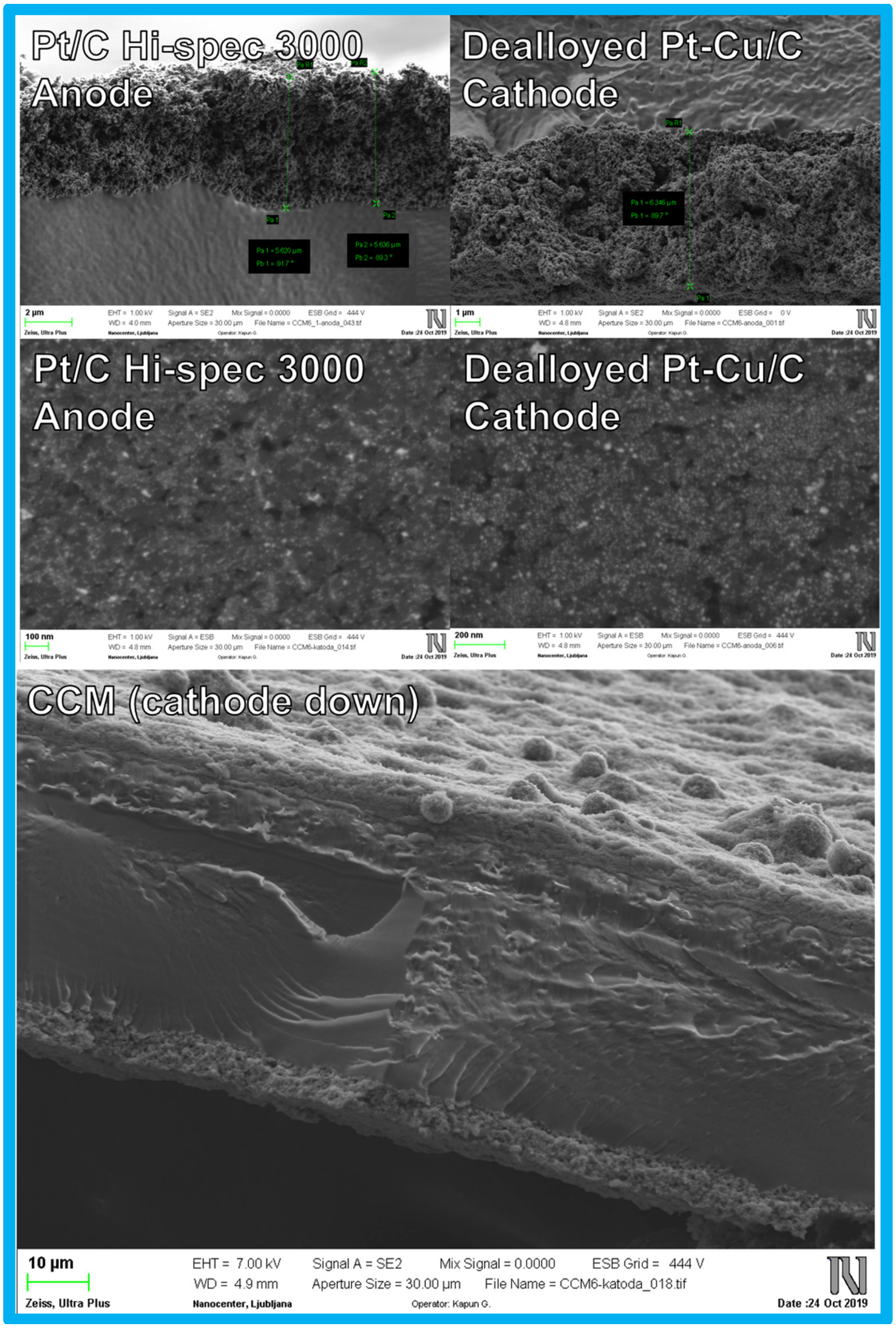

Figure S8: Cross-section SEM analysis of in-house fabricated CCM with 0.125 mg_{Pt} cm^{-2} dealloyed Pt-Cu/C cathode, 0.05 mg_{Pt} cm^{-2} Pt/C Hi-spec 3000 anode and ~50 µm membrane (NAFION NM 212, DuPont).

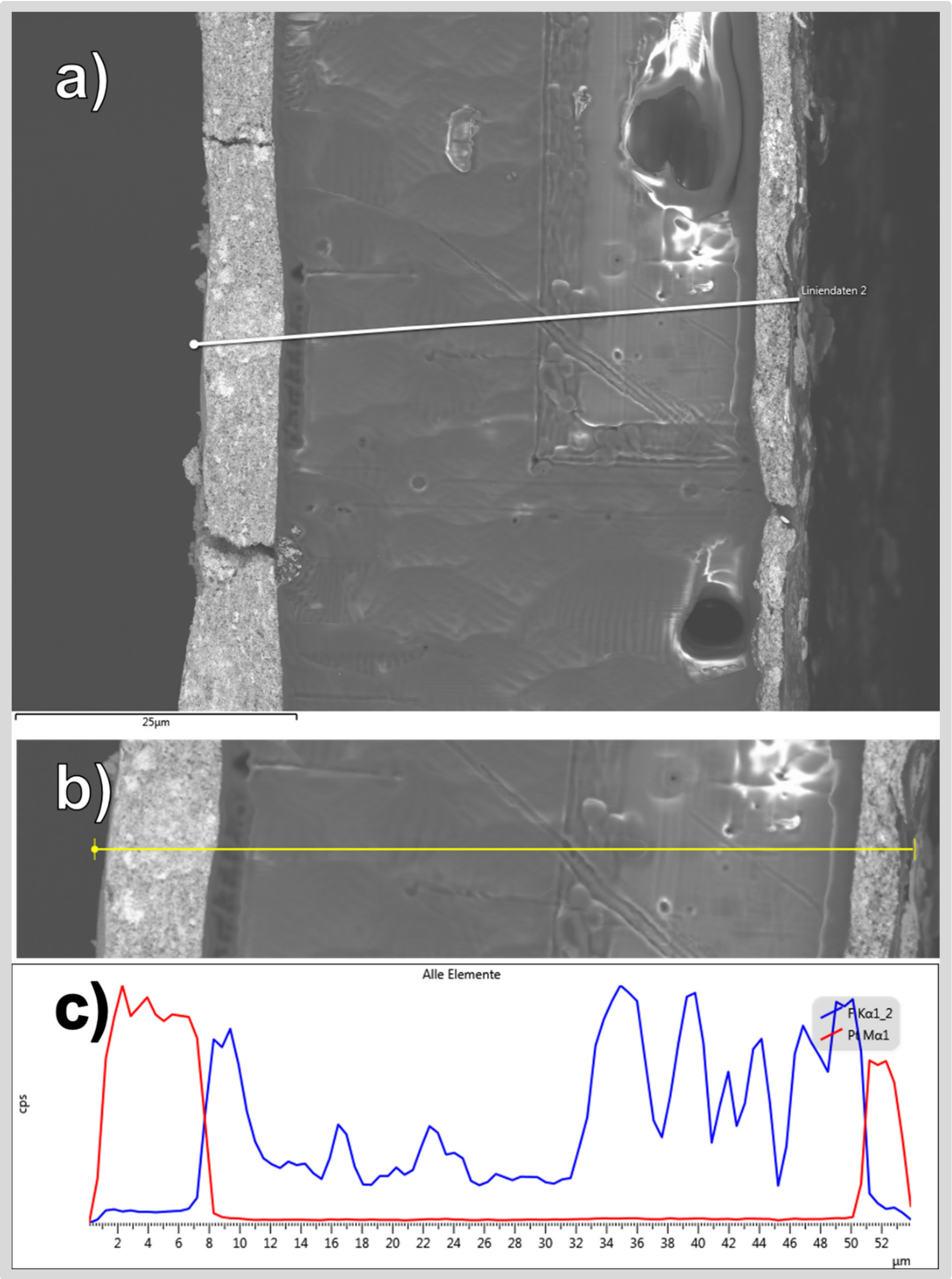

Figure S9: **(a)** Cross-section SEM analysis of commercially available CCM-H25-N212 (Quintech) with 0.6 mg_{Pt} cm^{-2} Pt/C cathode, 0.3 mg_{Pt} cm^{-2} Pt/C anode and ~50 µm membrane (NAFION NM 212, DuPont). **(b)** shows the area used for **(c)** EDX line scan analysis.

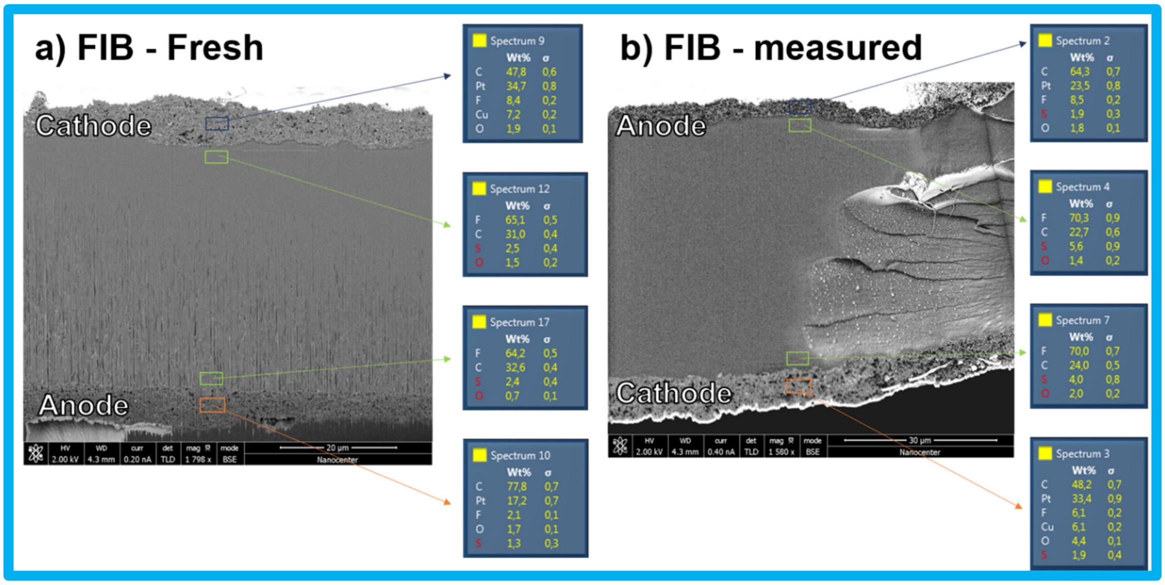

Figure S10: Semi-quantitative EDX analysis of the cryo-cut and FIB polished cross-sections of CCMs with dealloyed Pt-Cu/C electrocatalyst on the cathode and Hi-spec 3000 electrocatalyst on the anode – **(d)** fresh CCM and **(e)** measured CCM.

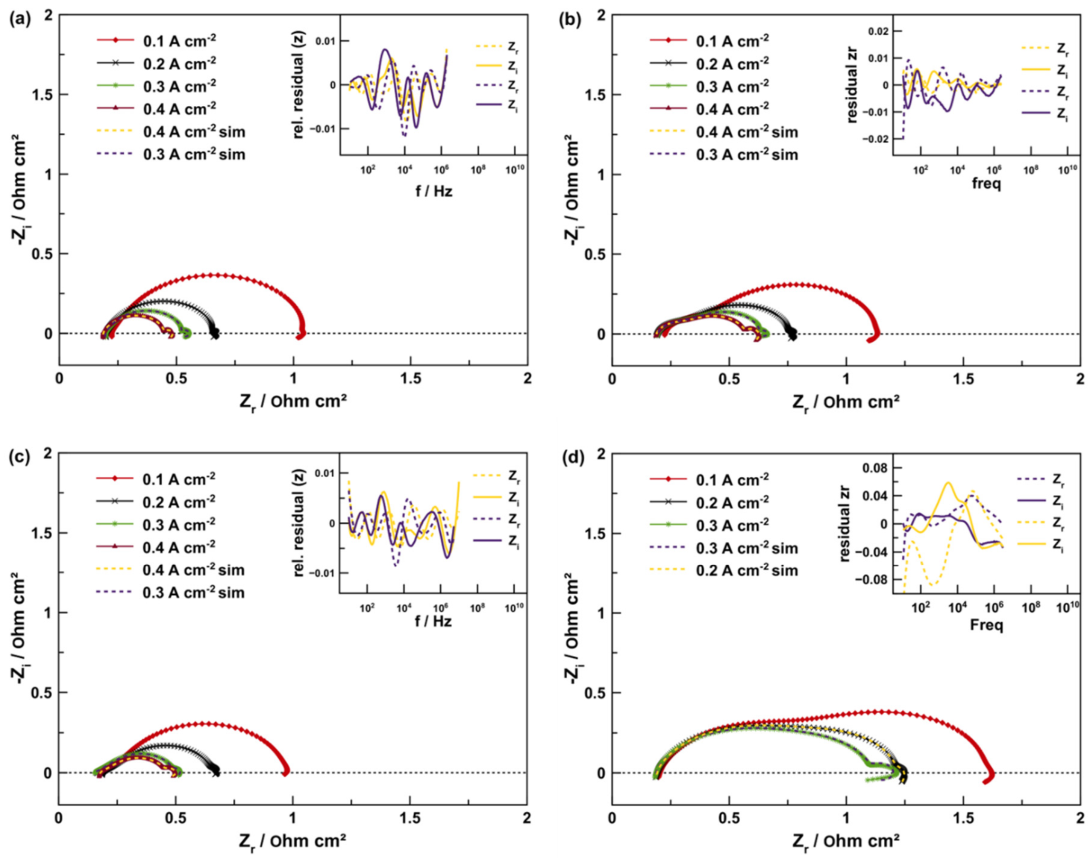

Figure S11: EIS measured from 0.4 to 0.1 A cm^{-2} under recorded with air/H_2 (600 mL min^{-1}) of CCMs Pt/Vul_0.8 **(a)** PtCu/KB_0.8 **(b)** Pt/Vul_0.6 **(c)** and PtCu/KB_0.6 **(d).** Simulation data is compared for selected points together with corresponding relative residuals.

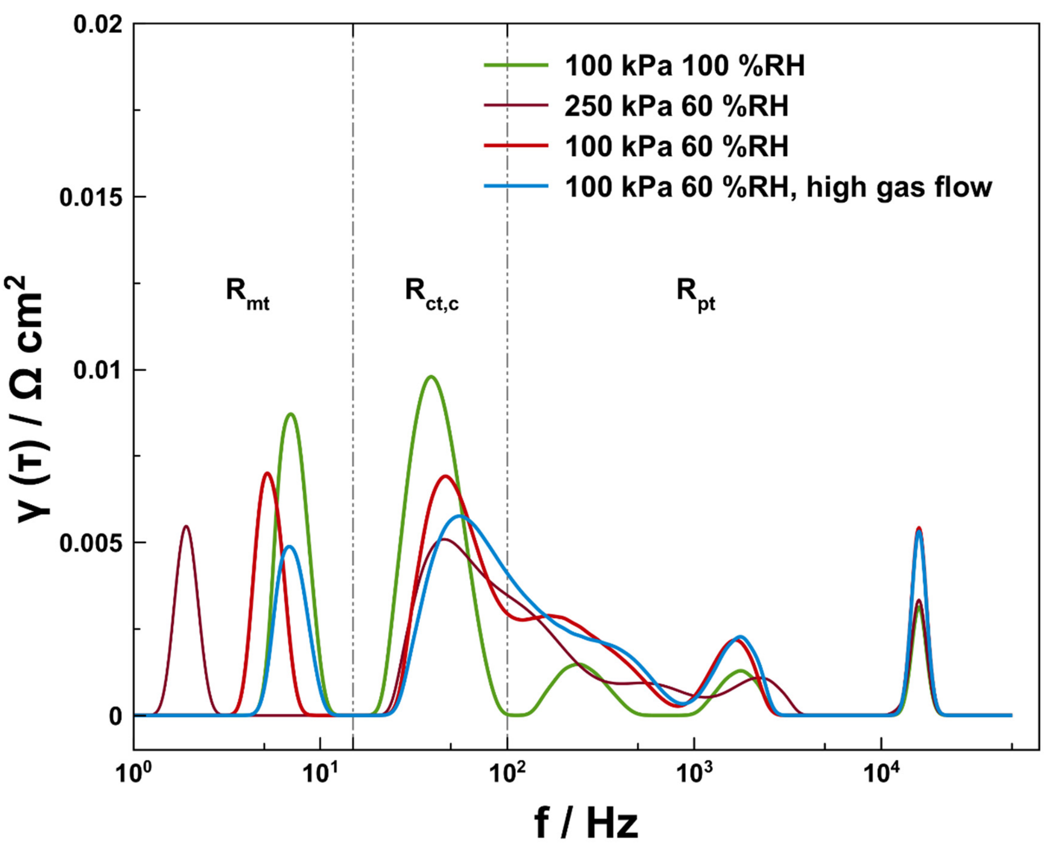

Figure S12: DRT analysis results for EIS recorded at varying operating conditions. Gas supply was set at 600/600 (standard) and 800/800 mL min^{-1} (high) of H_2/SA.

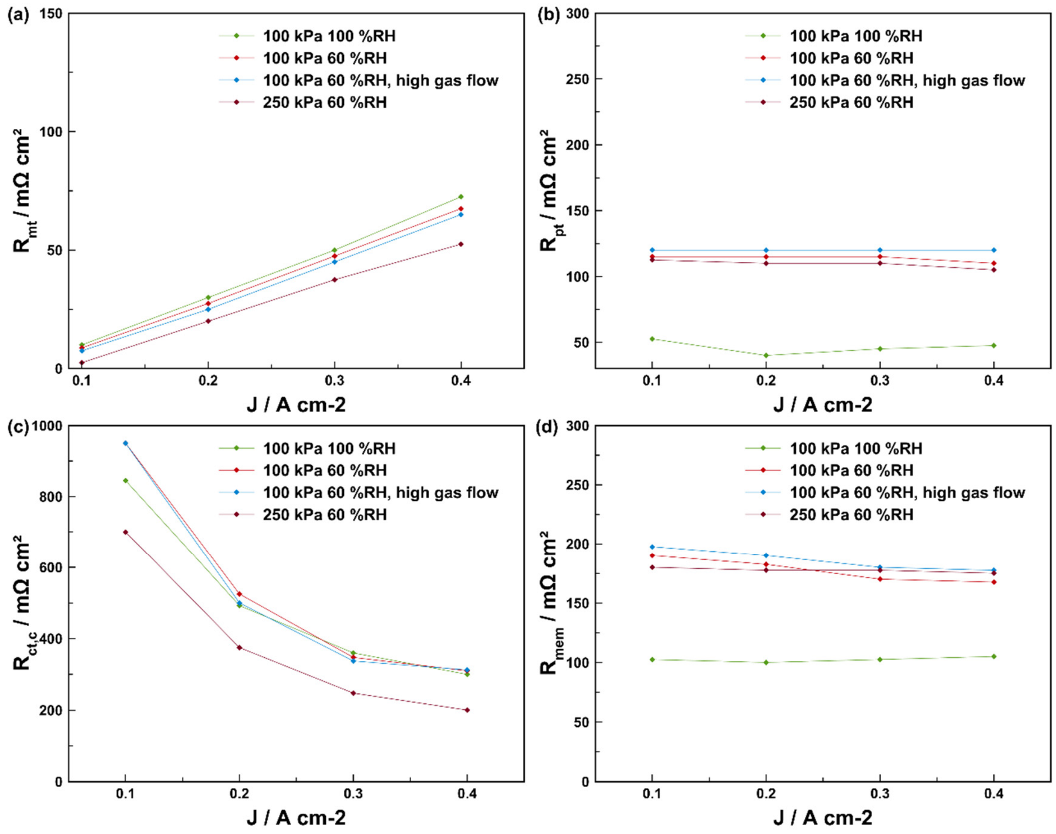

Figure S13: Resistance contributions R_{mt} **(a)**, R_{pt} **(b)** $R_{ct,c}$ **(c)** and R_{mem} **(d)** calculated from EIS at varying operating conditions. Gas supply was set at 600/600 (standard) and 800/800 mL min^{-1} (high) of H_2/SA.

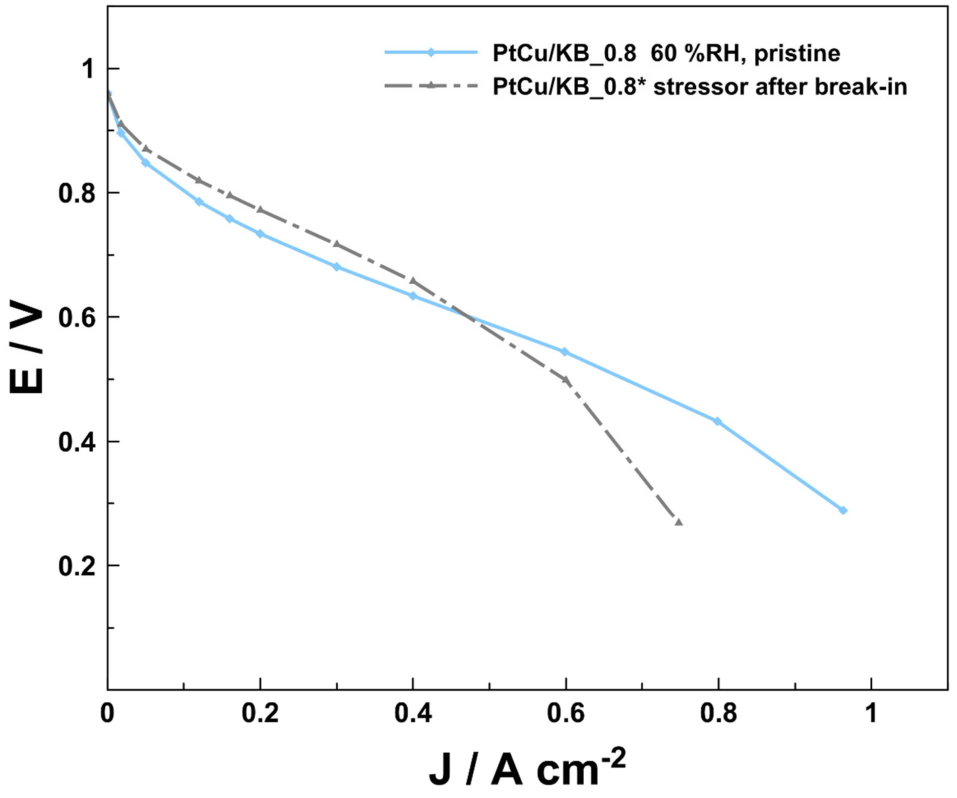

Figure S14: Polarization curve performed as stressor at 100 %RH 80 °C, 250 kPa H_2/air, 600 mL min^{-1} fixed flow.

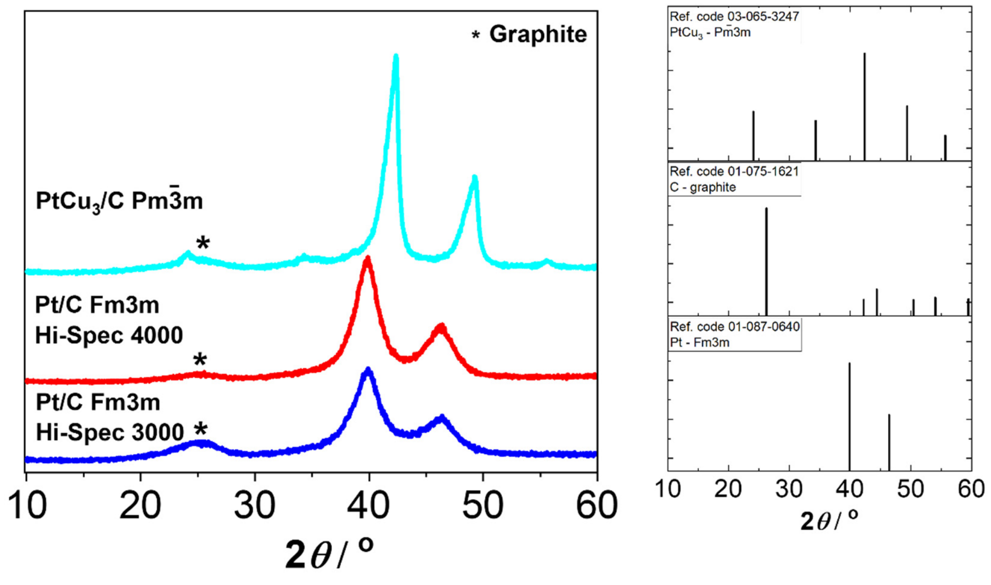

Figure S15: XRD Plots of the three compared catalysts and corresponding JCPDS card numbers.

Table S1: TF-RDE data of electrocatalysts used for fabrication of in-house CCMs.

Sample	$m_{e\text{-}cat}$ [µg]	Pt [wt%]	CO-area [cm^2]	$ECSA_{CO}$ [m^2 g^{-1}$_{Pt}$]	SA @ 0.9 V_{RHE} [mA cm^{-2}$_{Pt}$]	MA @ 0.9 V_{RHE} [A mg^{-1}$_{Pt}$]	SA @ 0.95 V_{RHE} [mA cm^{-2}$_{Pt}$]	MA @ 0.95 V_{RHE} [A mg^{-1}$_{Pt}$]
Pt/C Hi-spec 3000	20	20	3.49	87.29	0.58	0.50	0.10	0.09
Pt/C Hi-spec 4000	20	40	4.28	53.47	0.64	0.34	0.11	0.06
Dealloyed Pt-Cu/C	20	26	3.02	58.16	2.79	1.62	0.46	0.27

Table S2: Overview of results for active layer thickness determination using cryo-cut SEM cross sections, analyzed with SEM. Each measurement was performed four times. For anodes, three samples from different CCMs were analyzed.

Active Layer (AL)	Average thickness of SEM cross section [µm]	% error over one AL	% error over three different AL
Cathode: Hi-spec 4000	4.4	5.7	-
Cathode: Hi-spec 3000	11.7	2.2	-
Cathode: Dealloyed Pt-Cu/C	8.4	1.9	-
Anodes: Hi-spec 3000	4.4	5.6	6.9
CCM-H25-N212	5.9	26.5	-

Table S3: Results of the electrocatalyst loading determination. Every process of coating a GDL was repeated 3 times with the same ink and then repeated using a freshly prepared ink.

Electrocatalyst	Mass difference achieved with 40 Layers [mg]	% error
Hi-spec 4000	5.8 ±0.4	7
Hi-spec 3000	5.4 ±0.4	8
Dealloyed Pt-Cu/C	5.8 ±0.2	3
